# Performance of General Surgical Procedures in Outpatient Settings Before and After Onset of the COVID-19 Pandemic

**DOI:** 10.1001/jamanetworkopen.2023.1198

**Published:** 2023-03-02

**Authors:** Omair A. Shariq, Katherine A. Bews, David A. Etzioni, Michael L. Kendrick, Elizabeth B. Habermann, Cornelius A. Thiels

**Affiliations:** 1 Department of Surgery, Mayo Clinic, Rochester, Minnesota; 2 Robert D. and Patricia E. Kern Center for the Science of Health Care Delivery, Mayo Clinic, Rochester, Minnesota; 3 Department of Surgery, Mayo Clinic, Phoenix, Arizona

## Abstract

**Importance:**

The American College of Surgeons (ACS) has advocated for the expansion of outpatient surgery to conserve limited hospital resources and bed capacity, while maintaining surgical throughput, during the COVID-19 pandemic.

**Objective:**

To investigate the association of the COVID-19 pandemic with outpatient scheduled general surgery procedures.

**Design, Setting, and Participants:**

This multicenter, retrospective cohort study analyzed data from hospitals participating in the ACS National Surgical Quality Improvement Program (ACS-NSQIP) from January 1, 2016, to December 31, 2019 (before COVID-19), and from January 1 to December 31, 2020 (during COVID-19). Adult patients (≥18 years of age) who underwent any 1 of the 16 most frequently performed scheduled general surgery operations in the ACS-NSQIP database were included.

**Main Outcomes and Measures:**

The primary outcome was the percentage of outpatient cases (length of stay, 0 days) for each procedure. To determine the rate of change over time, multiple multivariable logistic regression models were used to assess the independent association of year with the odds of outpatient surgery.

**Results:**

A total of 988 436 patients were identified (mean [SD] age, 54.5 [16.1] years; 574 683 women [58.1%]), of whom 823 746 underwent scheduled surgery before COVID-19 and 164 690 had surgery during COVID-19. On multivariable analysis, the odds of outpatient surgery during COVID-19 (vs 2019) were higher in patients who underwent mastectomy for cancer (odds ratio [OR], 2.49 [95% CI, 2.33-2.67]), minimally invasive adrenalectomy (OR, 1.93 [95% CI, 1.34-2.77]), thyroid lobectomy (OR, 1.43 [95% CI, 1.32-1.54]), breast lumpectomy (OR, 1.34 [95% CI, 1.23-1.46]), minimally invasive ventral hernia repair (OR, 1.21 [95% CI, 1.15-1.27]), minimally invasive sleeve gastrectomy (OR, 2.56 [95% CI, 1.89-3.48]), parathyroidectomy (OR, 1.24 [95% CI, 1.14-1.34]), and total thyroidectomy (OR, 1.53 [95% CI, 1.42-1.65]). These odds were all greater than those observed for 2019 vs 2018, 2018 vs 2017, and 2017 vs 2016, suggesting that an accelerated increase in outpatient surgery rates in 2020 occurred as a consequence of COVID-19, rather than a continuation of secular trends. Despite these findings, only 4 procedures had a clinically meaningful (≥10%) overall increase in outpatient surgery rates during the study period: mastectomy for cancer (+19.4%), thyroid lobectomy (+14.7%), minimally invasive ventral hernia repair (+10.6%), and parathyroidectomy (+10.0%).

**Conclusions and Relevance:**

In this cohort study, the first year of the COVID-19 pandemic was associated with an accelerated transition to outpatient surgery for many scheduled general surgical operations; however, the magnitude of percentage increase was small for all but 4 procedure types. Further studies should explore potential barriers to the uptake of this approach, particularly for procedures that have been shown to be safe when performed in an outpatient setting.

## Key Points

**Question:**

Was the COVID-19 pandemic associated with an accelerated transition from inpatient to outpatient general surgery procedures in hospitals participating in the American College of Surgeons National Surgical Quality Improvement Program?

**Findings:**

In this cohort study of 988 436 patients who underwent 16 common general surgery operations, the onset of the COVID-19 pandemic was associated with a significantly increased odds of outpatient surgery for 8 procedures (above and beyond secular trends). However, the actual percentage increase in outpatient cases was small (ie, <10%) for all but 4 procedures.

**Meaning:**

These findings suggest that despite calls for the expansion of outpatient surgery to mitigate the growing backlog of surgical cases in the wake of the COVID-19 pandemic, uptake of this practice occurred in only a small subset of operations.

## Teaser

This cohort study assesses trends in outpatient surgery rates across a range of commonly scheduled general surgery procedures during a 5-year period, including the start of the COVID-19 pandemic.

## Introduction

Widespread diffusion of minimally invasive technologies and advances in perioperative care have substantially reduced the length of time that patients spend in the hospital recovering from surgery. In the current climate of value-based care, surgical procedures are increasingly performed in an outpatient setting. The adoption of outpatient surgery has been pervasive across surgical subspecialties, with studies describing the feasibility and safety of outpatient procedures such as colectomy,^1,2^ inguinal hernia repair,^3^ and adrenalectomy.^4^ Benefits of same-day discharge include a reduced risk of exposure to nosocomial infections, cost savings due to reduced use of resources, and increased patient satisfaction due to the ability to convalesce in familiar surroundings.^5^

An additional impetus for outpatient surgery has arisen in the era of COVID-19, due to the dramatic shift in use of health care resources that ensued in the wake of the pandemic. It is estimated that more than 28 million adult operations were canceled or postponed worldwide during the peak of the pandemic in 2020, as hospitals curtailed elective surgical procedures to preserve personal protective equipment, reduce viral transmission, and free up bed capacity and personnel.^6^ Importantly, while the impact of COVID-19 surges on hospitalizations may have lessened in 2022, ongoing staffing shortages and a growing backlog of surgical cases at many institutions across the country indicate an ongoing need to maximize resources.

Competing priorities between managing the COVID-19 crisis and providing nonurgent (but necessary) surgical treatment for patients led to the release of guidelines from professional societies, such as the American College of Surgeons (ACS)^7^ and other organizations,^8^ to assist in the triage of cases for the safe reintroduction of scheduled surgery. These guidelines recognize that postoperative inpatient admission uses key hospital resources that need to be allocated toward the care of acutely unwell patients with COVID-19 and exposes patients undergoing routine surgery to the risk of nosocomial COVID-19 infection. A proposed strategy for mitigating these issues while maintaining surgical volume includes the expansion of outpatient surgery^9^; however, there is a paucity of data regarding the uptake of this practice in US hospitals before and after the start of the pandemic.

We hypothesized that the advent of COVID-19 led to an accelerated transition from inpatient to outpatient scheduled general surgery in hospitals participating in the ACS National Surgical Quality Improvement Program (ACS-NSQIP). To test this hypothesis, we performed a retrospective analysis of the ACS-NSQIP database to assess trends in outpatient surgery rates across a range of commonly scheduled general surgery procedures over a 5-year period, including the start of the COVID-19 pandemic. We were specifically interested in whether an observed increase in the odds of outpatient surgery during the pandemic in 2020 went above and beyond overall secular trends observed from 2016 to 2019.

## Methods

### Data Source and Study Cohort

This cohort study was determined to be exempt from review and informed consent by the institutional review board of the Mayo Clinic, Rochester, Minnesota, owing to the use of deidentified data. The study followed the Strengthening the Reporting of Observational Studies in Epidemiology (STROBE) reporting guideline.

Adult patients (ie, patients aged ≥18 years) who underwent 1 of 16 commonly scheduled general surgery operations (minimally invasive colectomy for cancer, minimally invasive colectomy for benign disease, lumpectomy for breast cancer, mastectomy for breast cancer, minimally invasive adrenalectomy, thyroid lobectomy, total thyroidectomy, parathyroidectomy, minimally invasive inguinal hernia repair, open inguinal hernia repair, minimally invasive ventral hernia repair, open umbilical hernia repair, minimally invasive sleeve gastrectomy, minimally invasive gastric bypass, minimally invasive cholecystectomy, and minimally invasive fundoplication) from January 1, 2016, to December 31, 2019 (before COVID-19), and January 1 to December 31, 2020 (during the COVID-19 pandemic), were identified in the ACS-NSQIP database using *Current Procedural Terminology* codes (eTable 1 in Supplement 1). These 16 procedures were selected as they represented the most frequently performed general surgery operations identified by the surgical specialty variable within the ACS-NSQIP database and consisted of a variety of procedures. To limit case-mix variation over time, each procedure group was limited to a consistent set of diagnosis codes specific to that procedure, based on codes from the *International Classification of Diseases, Ninth Revision, Clinical Modification*, or the *International Statistical Classification of Diseases, Tenth Revision, Clinical Modification* (eTable 1 in Supplement 1). Patients with severe preoperative comorbidities that were likely to necessitate an inpatient stay (preoperative ventilator dependence, sepsis, septic shock, systemic inflammatory response syndrome, open and/or infected wound, acute renal failure, >4 U of red blood cell transfused within 72 hours prior to procedure, American Society of Anesthesiologists [ASA] class V, and disseminated cancer), and urgent or emergent operations were excluded from the analysis. Details regarding the number of hospitals participating in the ACS-NSQIP, the total number of cases submitted, the process for data collection, definitions of outcome variables, and procedures for ensuring the reliability of the data are described in the ACS-NSQIP Participant Use Data File user guide.^10^

### Covariates and Outcome Definitions

Patient demographic and clinical covariates recorded included age, sex, race and ethnicity, body mass index, ASA classification (I: normal healthy patient; II: mild systemic disease; III: severe systemic disease; and IV: severe systemic disease that is a constant threat to life), and functional status (classified as independent, partially dependent, and totally dependent). Patient-level comorbidity variables included diabetes (using oral medications or insulin), hypertension, smoking status (current smoker within 1 year), congestive heart failure, severe chronic obstructive pulmonary disease, and chronic corticosteroid use. Patients of the following racial and ethnic groups were compared as a demographic variable and to investigate unexplained variations in use of surgery: Black or African American (hereinafter *Black*), Hispanic White, non-Hispanic White, or other or unknown. The other or unknown category consisted of patients reported in ACS-NSQIP as American Indian or Alaska Native, Asian, Native Hawaiian or other Pacific Islander, some other race, or unknown. Hospitals participating in ACS-NSQIP enter data regarding race and ethnicity based on the patient’s self-reported information, or these variables are assigned by institutional personnel per internal practices as per the medical record.

The primary outcome measure was the odds of outpatient surgery for each procedure per year and whether the COVID-19 pandemic in 2020 was associated with an increased odds of outpatient surgery compared with prior years. We applied the International Association for Ambulatory Surgery definition of an outpatient procedure as being a planned procedure performed on a patient who is discharged on the same calendar day (ie, postoperative day 0).^11^

### Statistical Analysis

Continuous variables are presented as mean (SD), and categorical data are presented as count (percentage). To determine whether there was a significant linear trend in the proportion of outpatient procedures performed at hospitals participating in the ACS-NSQIP over time, the Cochran-Armitage test was performed. In addition to a statistically significant trend in outpatient cases over time (defined as 2-tailed *P* < .05), we considered a clinically meaningful increase to be a 10% or greater increase in the proportion of outpatient procedures from the beginning of the study period to December 31, 2020. To determine the rate of change over time (and whether the COVID-19 pandemic in 2020 further accelerated the transition toward outpatient surgery), multiple univariate logistic regression models stratified by procedure type were used to assess the association between year of operation and the odds of outpatient surgery. Multiple multivariable logistic regression models adjusting for age, sex, ASA class, smoking status, and body mass index were also developed to assess the independent association of year with the odds of outpatient surgery. All statistical analyses were performed using SAS software, version 9.4 (SAS Institute Inc), and R Studio, version 4.1.2 (R Project for Statistical Computing).

## Results

### Overall Patient Cohort

A total of 988 436 patients were identified (mean [SD] age, 54.5 [16.1] years; 574 683 women [58.1%], 413 727 men [41.9%], and 26 nonbinary or not reported [0.003%]). In terms of race and ethnicity, 100 788 were Black (10.2%), 65 298 were Hispanic (6.6%), and 623 587 were non-Hispanic White (63.1%). Overall, 823 746 patients underwent scheduled surgery before COVID-19 and 164 690 underwent surgery during the COVID-19 pandemic. The mean (SD) age of the patients who underwent surgery before COVID-19 was 54.3 (16.1) years compared with 55.1 (16.1) years after the onset of the pandemic. Most patients in both groups were female (58.2% before COVID-19 vs 58.0% during COVID-19) and non-Hispanic White (64.1% before COVID-19 vs 57.8% during COVID-19). The most frequently performed elective general surgical procedures at ACS-NSQIP–participating hospitals before COVID-19 were minimally invasive cholecystectomy (21.0%), open inguinal hernia repair (10.4%), and open umbilical hernia repair (8.0%). During the COVID-19 pandemic, the most frequently performed operations were minimally invasive cholecystectomy (22.7%), open inguinal hernia repair (9.3%), and minimally invasive colectomy for cancer (8.8%). Detailed demographics and preoperative variables are shown in Table 1.

**Table 1.** Baseline Characteristics of Patients Who Underwent Surgery Before and During the COVID-19 Pandemic^a^

Characteristic	Patient cohort	
	Before the COVID-19 pandemic^b^ (n = 823 746)	During the COVID-19 pandemic^c^ (n = 164 690)
Age, mean (SD), y	54.3 (16.1)	55.1 (16.1)
Sex		
Men	344 534 (41.8)	69 193 (42.0)
Women	479 200 (58.2)	95 483 (58.0)
Nonbinary	11 (0.001)	14 (0.01)
Not reported	1 (0.0001)	0
Race and ethnicity		
Black or African American	84 342 (10.2)	16 446 (10.0)
Hispanic White	53 321 (6.5)	11 977 (7.3)
Non-Hispanic White	528 356 (64.1)	95 231 (57.8)
Other or unknown^d^	157 727 (19.1)	41 036 (24.9)
BMI		
<18.5	6556 (0.8)	1509 (0.9)
18.5-24.9	160 860 (19.5)	33 536 (20.4)
25.0-29.9	243 610 (29.6)	51 109 (31.0)
≥30.0	404 659 (49.1)	76 162 (46.2)
Not reported	8061 (1.0)	2374 (1.4)
ASA class		
I-II	490 003 (59.5)	97 409 (59.1)
III-IV	332 721 (40.4)	67 078 (40.7)
Not reported	1022 (0.1)	203 (0.1)
Functional status		
Independent	811 682 (98.5)	162 560 (98.7)
Partially dependent	5221 (0.6)	1039 (0.6)
Totally dependent	688 (0.1)	127 (0.1)
Not reported	6155 (0.7)	964 (0.6)
Comorbidities		
Diabetes	115 148 (14.0)	22 097 (13.4)
Current smoker	118 679 (14.4)	21 722 (13.2)
Severe COPD	23 702 (2.9)	4393 (2.7)
Chronic steroid use	22 498 (2.7)	4975 (3.0)
Hypertension	334 454 (40.6)	65 068 (39.5)
Procedure		
Minimally invasive colectomy		
For cancer	63 300 (7.7)	14 503 (8.8)
For benign disease	35 058 (4.3)	7900 (4.8)
Breast lumpectomy	58 323 (7.1)	14 308 (8.7)
Mastectomy	44 063 (5.3)	10 603 (6.4)
Minimally invasive cholecystectomy	173 280 (21.0)	37 315 (22.7)
Minimally invasive inguinal hernia repair	57 544 (7.0)	13 526 (8.2)
Open inguinal hernia repair	86 042 (10.4)	15 251 (9.3)
Minimally invasive ventral hernia repair	63 771 (7.7)	13 144 (8.0)
Open umbilical hernia repair	66 207 (8.0)	10 861 (6.6)
Minimally invasive fundoplication	7235 (0.9)	1299 (0.8)
Minimally invasive sleeve gastrectomy	55 578 (6.7)	4571 (2.8)
Minimally invasive gastric bypass	25 781 (3.1)	2868 (1.7)
Parathyroidectomy	21 750 (2.6)	4595 (2.8)
Thyroid lobectomy	22 225 (2.7)	4774 (2.9)
Total thyroidectomy	40 298 (4.9)	8366 (5.1)
Minimally invasive adrenalectomy	3291 (0.4)	806 (0.5)

Abbreviations: ASA, American Society of Anesthesiologists; BMI, body mass index (calculated as weight in kilograms divided by height in meters squared); COPD, chronic obstructive pulmonary disease.

^a^ Unless otherwise indicated, data are expressed as No. (%) of patients. Percentages have been rounded and may not total 100.

^b^ Defined as the period spanning January 1, 2016, to December 31, 2019.

^c^ Defined as the period spanning January 1 to December 31, 2020.

^d^ Includes American Indian or Alaska Native, Asian, Native Hawaiian or other Pacific Islander, other race or ethnicity, or unknown race or ethnicity.

### Trends in Outpatient Surgery During the Overall Study Period

From 2016 to 2020, a linear trend was indicative of a significant increase in the proportion of outpatient cases for 10 of 16 procedures (Figure and eFigure in Supplement 1). Despite statistical significance, however, only 4 of these 10 procedures had a clinically meaningful (≥10%) increase in the proportion of outpatient operations during the 5-year period: mastectomy for breast cancer (9.2% to 28.6%; +19.4%), thyroid lobectomy (43.2% to 57.9%; +14.7%), minimally invasive ventral hernia repair (58.8% to 69.4%; +10.6%), and parathyroidectomy (51.8% to 61.8%; +10.0%) (Figure). The 6 procedures for which the proportion of outpatient cases significantly trended upward from 2016 to 2020, but did not exceed a 10% increase from baseline, included total thyroidectomy (13.8% to 22.6%; +8.8%), minimally invasive adrenalectomy (2.5% to 10.8%; +8.3%), minimally invasive fundoplication (7.4% to 11.7%; +4.3%), breast lumpectomy (89.6% to 93.3%; +3.7%), minimally invasive colectomy for cancer (0.8% to 1.3%; +0.5%), and minimally invasive sleeve gastrectomy (0.8% to 2.3%; +1.5%) (eFigure in Supplement 1). A very small (but significant) decreased trend in outpatient cases from 2016 to 2020 was observed for 2 procedures: open inguinal hernia repair (89.6% to 89.1%; −0.5%) and open umbilical hernia repair (92.6% to 92.1%; −0.5%) (eFigure in Supplement 1). The remaining 4 procedures for which there was no significant change in the proportion of outpatient cases over the course of the entire study period included minimally invasive inguinal hernia repair (92.2% to 92.7%; +0.5%), minimally invasive gastric bypass (0.6% to 0.8%; +0.2%), minimally invasive cholecystectomy (65.4% to 64.8%; −0.6%), and minimally invasive colectomy for benign disease (0.4% to 0.3%; −0.1%) (eFigure in Supplement 1).

**Figure.**
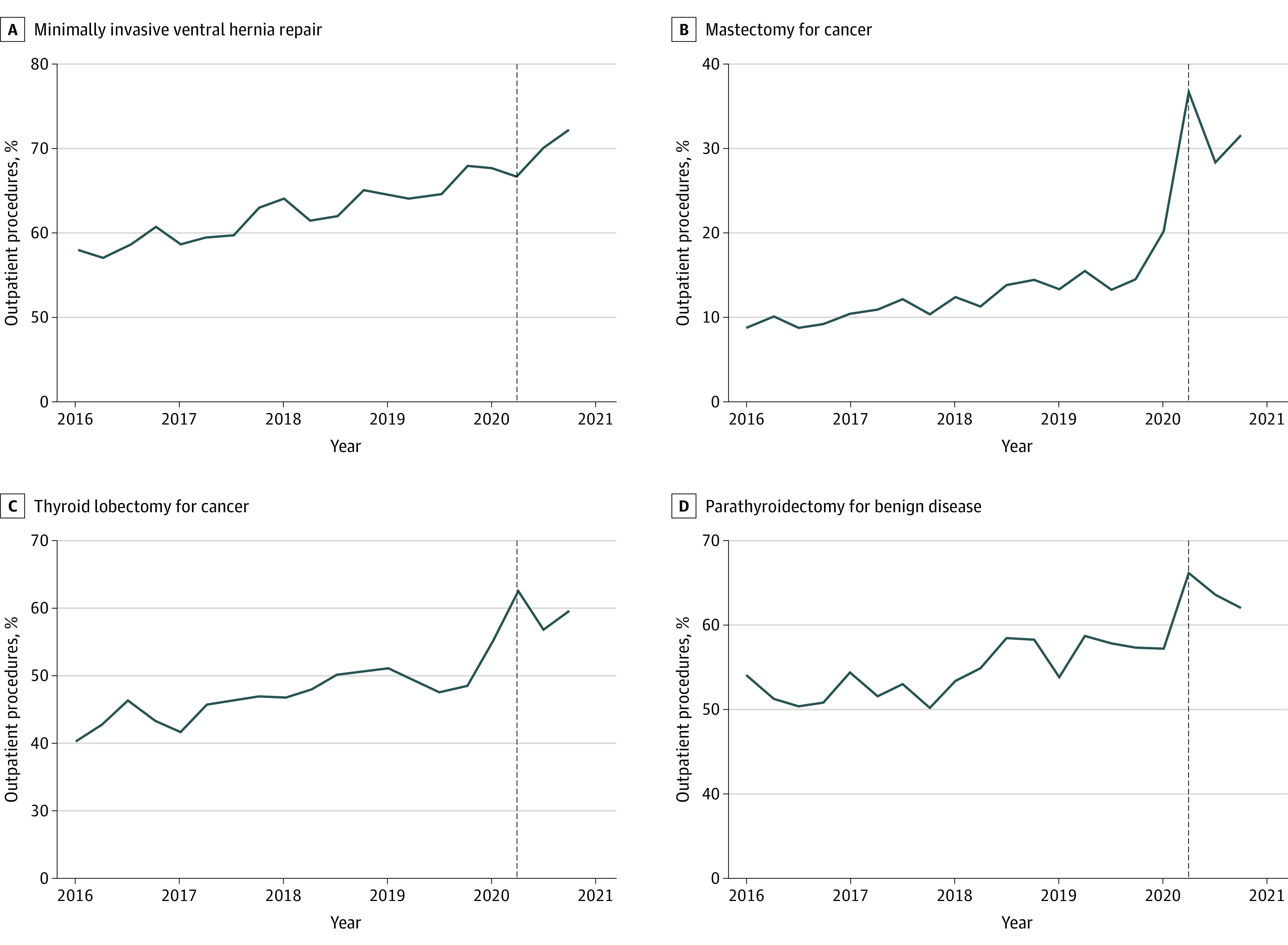
Outpatient Procedure Percentage From 2016 to 2020 Stratified by Procedure Type Trend lines indicate significant (*P* < .05) trends over time as determined by Cochran-Armitage tests. The vertical dashed lines represent quarter 2 of 2020 (the onset of the COVID-19 pandemic in the US). Minimally invasive ventral hernia repair increased from 58.8% to 69.4% (+10.6%); mastectomy for breast cancer, from 9.2% to 28.6% (+19.4%); thyroid lobectomy, from 43.2% to 57.9% (+14.7%); and parathyroidectomy, from 51.8% to 61.8% (+10.0%).

### Association of the COVID-19 Pandemic With the Odds of Undergoing Outpatient Surgery

The unadjusted odds of undergoing outpatient surgery in 2020 vs 2019 were significantly higher in patients who underwent the following 8 procedures: mastectomy for breast cancer (odds ratio [OR], 2.44 [95% CI, 2.28-2.61]), minimally invasive adrenalectomy (OR, 1.96 [95% CI, 1.37-2.81), thyroid lobectomy (OR, 1.43 [95% CI, 1.32-1.55]), breast lumpectomy (OR, 1.35 [95% CI, 1.24-1.47]), minimally invasive ventral hernia repair (OR, 1.21 [95% CI, 1.15-1.27]), minimally invasive sleeve gastrectomy (OR, 2.52 [95% CI, 1.86-3.40]), parathyroidectomy (OR, 1.22 [95% CI, 1.13-1.32]), and total thyroidectomy (OR, 1.51 [95% CI, 1.40-1.62]) (all *P* < .001) (Table 2). These odds were all greater than those observed for 2019 vs 2018, 2018 vs 2017, and 2017 vs 2016, suggesting that an additional increase in outpatient surgery rates for these procedures in 2020 occurred as a consequence of COVID-19 rather than secular trends. These findings were confirmed on multivariable analysis, which again showed a significantly higher odds of undergoing outpatient surgery in 2020 vs 2019 for the aforementioned procedures: mastectomy for breast cancer (OR, 2.49 [95% CI, 2.33-2.67]), minimally invasive adrenalectomy (OR, 1.93 [95% CI, 1.34-2.77]), thyroid lobectomy (OR, 1.43 [95% CI, 1.32-1.54]), breast lumpectomy (OR, 1.34 [95% CI, 1.23-1.46]), minimally invasive ventral hernia repair (OR, 1.21 [95% CI, 1.15-1.27]), minimally invasive sleeve gastrectomy (OR, 2.56 [95% CI, 1.89-3.48]), parathyroidectomy (OR, 1.24 [95% CI, 1.14-1.34]), and total thyroidectomy (OR, 1.53 [95% CI, 1.42-1.65]) (all *P* ≤ .001) (Table 2). We further confirmed these findings in a univariate and multivariable sensitivity analysis of cases performed during the second to fourth quarters of 2020 (ie, after the COVID-19 pandemic was declared a national emergency in the US) vs the second to fourth quarters of 2019 (eTable 2 in Supplement 1).

**Table 2.** Odds of Undergoing Outpatient General Surgery Procedures Before vs During the COVID-19 Pandemic

Procedure	Year	Univariate OR (95% CI)	*P* value	Multivariable adjusted OR (95% CI)^a^	*P* value
Minimally invasive colectomy for cancer	2017 vs 2016	1.15 (0.89-1.47)	.29	1.15 (0.89-1.48)	.28
	2018 vs 2017	1.08 (0.86-1.36)	.51	1.09 (0.87-1.38)	.47
	2019 vs 2018	1.14 (0.92-1.41)	.22	1.16 (0.94-1.44)	.17
	2020 vs 2019	1.19 (0.97-1.45)	.10	1.21 (0.99-1.48)	.07
Lumpectomy for cancer	2017 vs 2016	1.10 (1.02-1.19)	.02	1.09 (1.00-1.18)	.04
	2018 vs 2017	1.01 (0.93-1.09)	.83	1.01 (0.93-1.09)	.81
	2019 vs 2018	1.08 (1.00-1.17)	.05	1.10 (1.02-1.19)	.02
	2020 vs 2019	1.35 (1.24-1.47)	<.001	1.34 (1.23-1.46)	<.001
Mastectomy for cancer	2017 vs 2016	1.22 (1.12-1.33)	<.001	1.22 (1.11-1.33)	<.001
	2018 vs 2017	1.21 (1.11-1.31)	<.001	1.21 (1.11-1.31)	<.001
	2019 vs 2018	1.10 (1.02-1.19)	.02	1.10 (1.02-1.19)	.02
	2020 vs 2019	2.44 (2.28-2.61)	<.001	2.49 (2.33-2.67)	.001
Minimally invasive adrenalectomy	2017 vs 2016	0.54 (0.25-1.14)	.11	0.51 (0.24-1.09)	.08
	2018 vs 2017	3.47 (1.76-6.84)	<.001	3.62 (1.83-7.15)	<.001
	2019 vs 2018	1.31 (0.85-2.01)	.22	1.31 (0.85-2.02)	.22
	2020 vs 2019	1.96 (1.37-2.81)	<.001	1.93 (1.34-2.77)	<.001
Thyroid lobectomy for cancer	2017 vs 2016	1.08 (1.00-1.17)	.04	1.09 (1.01-1.18)	.03
	2018 vs 2017	1.16 (1.08-1.25)	<.001	1.16 (1.08-1.25)	<.001
	2019 vs 2018	1.01 (0.94-1.08)	.85	1.01 (0.93-1.08)	.88
	2020 vs 2019	1.43 (1.32-1.55)	<.001	1.43 (1.32-1.54)	<.001
Minimally invasive inguinal hernia repair	2017 vs 2016	1.03 (0.94-1.13)	.51	1.07 (0.97-1.18)	.16
	2018 vs 2017	1.07 (0.98-1.17)	.12	1.09 (0.99-1.19)	.07
	2019 vs 2018	0.93 (0.86-1.02)	.11	0.93 (0.85-1.02)	.11
	2020 vs 2019	1.04 (0.96-1.14)	.36	1.04 (0.96-1.14)	.34
Open inguinal hernia repair	2017 vs 2016	0.98 (0.92-1.04)	.52	1.01 (0.95-1.08)	.76
	2018 vs 2017	0.97 (0.91-1.03)	.29	0.97 (0.91-1.04)	.40
	2019 vs 2018	0.93 (0.87-0.99)	.02	0.93 (0.88-0.99)	.03
	2020 vs 2019	1.07 (1.00-1.14)	.05	1.08 (1.01-1.15)	.04
Minimally invasive ventral hernia repair	2017 vs 2016	1.07 (1.02-1.12)	.005	1.07 (1.02-1.12)	.006
	2018 vs 2017	1.13 (1.08-1.18)	<.001	1.14 (1.09-1.20)	<.001
	2019 vs 2018	1.09 (1.05-1.14)	<.001	1.11 (1.06-1.17)	<.001
	2020 vs 2019	1.21 (1.15-1.27)	<.001	1.21 (1.15-1.27)	<.001
Open umbilical hernia repair	2017 vs 2016	1.02 (0.94-1.11)	.61	1.02 (0.93-1.11)	.71
	2018 vs 2017	0.91 (0.84-0.99)	.02	0.93 (0.85-1.01)	.07
	2019 vs 2018	1.00 (0.92-1.08)	.94	1.01 (0.93-1.10)	.79
	2020 vs 2019	1.01 (0.92-1.10)	.85	1.00 (0.91-1.10)	.95
Minimally invasive sleeve gastrectomy	2017 vs 2016	0.92 (0.72-1.17)	.49	0.92 (0.72-1.16)	.47
	2018 vs 2017	0.98 (0.75-1.28)	.90	0.99 (0.76-1.29)	.92
	2019 vs 2018	1.29 (0.95-1.76)	.11	1.25 (0.92-1.71)	.16
	2020 vs 2019	2.52 (1.86-3.40)	<.001	2.56 (1.89-3.48)	<.001
Minimally invasive gastric bypass	2017 vs 2016	0.81 (0.52-1.26)	.35	0.80 (0.52-1.25)	.33
	2018 vs 2017	1.15 (0.71-1.87)	.56	1.14 (0.71-1.85)	.59
	2019 vs 2018	1.09 (0.63-1.87)	.76	1.07 (0.62-1.84)	.80
	2020 vs 2019	1.40 (0.79-2.51)	.25	1.30 (0.72-2.36)	.38
Parathyroidectomy	2017 vs 2016	1.03 (0.95-1.11)	.50	1.05 (0.97-1.14)	.23
	2018 vs 2017	1.16 (1.08-1.26)	<.001	1.15 (1.06-1.25)	<.001
	2019 vs 2018	1.03 (0.96-1.12)	.38	1.04 (0.96-1.12)	.32
	2020 vs 2019	1.22 (1.13-1.32)	<.001	1.24 (1.14-1.34)	<.001
Minimally invasive cholecystectomy	2017 vs 2016	1.05 (1.02-1.08)	<.001	1.06 (1.03-1.09)	<.001
	2018 vs 2017	1.00 (0.97-1.03)	.97	1.01 (0.98-1.04)	.73
	2019 vs 2018	0.99 (0.97-1.02)	.66	1.00 (0.97-1.02)	.74
	2020 vs 2019	0.93 (0.91-0.96)	<.001	0.94 (0.91-0.97)	<.001
Minimally invasive fundoplication	2017 vs 2016	0.95 (0.73-1.22)	.66	0.96 (0.74-1.24)	.75
	2018 vs 2017	0.98 (0.76-1.26)	.86	0.98 (0.76-1.27)	.87
	2019 vs 2018	1.48 (1.16-1.88)	.001	1.52 (1.20-1.94)	<.001
	2020 vs 2019	1.22 (0.97-1.53)	.10	1.20 (0.95-1.52)	.12
Minimally invasive colectomy for benign disease	2017 vs 2016	1.17 (0.73-1.89)	.52	1.19 (0.74-1.92)	.47
	2018 vs 2017	0.66 (0.41-1.09)	.10	0.64 (0.39-1.06)	.08
	2019 vs 2018	0.91 (0.53-1.55)	.72	0.90 (0.52-1.55)	.70
	2020 vs 2019	1.25 (0.73-2.13)	.42	1.35 (0.79-2.31)	.28
Total thyroidectomy for cancer	2017 vs 2016	0.97 (0.90-1.05)	.48	0.98 (0.90-1.06)	.56
	2018 vs 2017	1.19 (1.10-1.29)	<.001	1.19 (1.10-1.29)	<.001
	2019 vs 2018	1.05 (0.97-1.13)	.26	1.05 (0.97-1.13)	.23
	2020 vs 2019	1.51 (1.40-1.62)	<.001	1.53 (1.42-1.65)	<.001

Abbreviation: OR, odds ratio.

^a^ Covariates adjusted for in multivariable logistic regression models included American Society of Anesthesiologists class, age, smoking status, sex, and body mass index.

## Discussion

The COVID-19 pandemic placed unprecedented strain on health care systems and necessitated alterations in the delivery of surgical care, including the consideration of same-day discharge for procedures that would traditionally have entailed inpatient admission. To our knowledge, this study is the first to investigate the association of the pandemic with the transition from inpatient to outpatient surgery across a broad range of commonly scheduled general surgical procedures. For most procedures, a statistically significant increase in the number of outpatient cases was observed over the last 5 years, with an accelerated transition toward same-day discharge seen after COVID-19 was declared a global pandemic in 2020. For all but 4 procedures, however, this did not translate into a clinically meaningful increase in the proportion of outpatient surgical procedures performed compared with prepandemic levels.

Mastectomy for breast cancer demonstrated the greatest increase in the proportion of outpatient procedures, both throughout the entire duration of the study and following the onset of the COVID-19 pandemic. These findings mirror the expansion of Enhanced Recovery After Surgery algorithms in breast surgery^12^ and may be explained by an increased use of liposomal bupivacaine field blocks and a shift toward prepectoral implant reconstruction, which is associated with significantly less pain than when the pectoralis muscle is disrupted. Prepandemic studies^13,14^ have shown no increase in short-term complications, including readmissions and reoperations, after same-day discharge in patients who underwent mastectomy (with and without implant reconstruction). Two recently published ACS-NSQIP studies^15,16^ have also examined the impact of COVID-19 on breast cancer surgery, with findings that corroborate our own. Rubenstein et al^15^ found that rates of same-day discharge increased from 2019 to 2020 for patients who underwent mastectomy with alloplastic, but not autologous reconstruction. Similarly, Chiang et al,^16^ who only included patients undergoing alloplastic reconstruction, found an increased rate of outpatient cases in 2020 and did not observe an increased risk of complications compared with inpatient cases. While our study did not stratify patients undergoing mastectomy based on the presence and type of reconstruction, it is likely that very few patients discharged on postoperative day 0 underwent autologous reconstruction, as these patients are typically admitted to hospital for flap monitoring after surgery.

Our finding of a significant positive trend in outpatient thyroid lobectomy cases over the duration of this study echoes a number of single-institution and registry-based studies that have shown same-day discharge is increasingly used and does not entail an elevated risk of complications such as neck hematoma, hypocalcemia, and recurrent laryngeal nerve injury in the postoperative period.^17,18^ The increased popularity of this approach may also be attributable to high rates of patient satisfaction^19^ and substantial cost savings (estimated at >$15 000 per case by 1 study).^20^ We also observed a positive trend in the proportion of outpatient parathyroidectomies performed from 2016 to 2020, which likely reflects the increased adoption of focused parathyroidectomy over bilateral neck explorations. Above and beyond the trend in increased outpatient thyroid lobectomies and parathyroidectomies seen over the course of this study, we also observed 43% and 24% increased odds, respectively, of same-day discharge for both of these procedures during the COVID-19 pandemic compared with 2019. This contrasts with the findings of a recently published survey of members of the American Association of Endocrine Surgeons,^21^ who reported no increase in the proportion of outpatient thyroid lobectomies and parathyroidectomies after the onset of the pandemic. This may be explained by most survey respondents being in specialized endocrine surgery practices, who were likely to have already been performing these procedures on an outpatient basis prior to the pandemic. Interestingly, respondents reported that they were more likely to discharge patients undergoing total thyroidectomy on the day of surgery during the pandemic. The findings of our study support this, as we observed a 51% increased odds of outpatient total thyroidectomy in 2020 compared with 2019. However, the raw percentage increase in outpatient total thyroidectomy cases from the beginning to the end of the study period was only 8.8% (from 13.8% in 2016 to 22.6% in 2020), which fell below our a priori defined threshold of at least 10% as a clinically meaningful rise in outpatient cases.

Although the safety and feasibility of outpatient minimally invasive ventral hernia repair has previously been demonstrated,^22^ to our knowledge, no published studies have evaluated the impact of the COVID-19 pandemic on elective ventral hernia repair practice patterns. Moreover, guidelines from the European Hernia Society on hernia surgery in adult patients during the pandemic do not discuss an outpatient approach.^23^ Nonetheless, our data show that in addition to a positive trend in outpatient minimally invasive ventral hernia repair rates in the 4 years that preceded the pandemic, onset of the COVID-19 pandemic in 2020 was associated with 21% increased odds of same-day discharge compared with patients who underwent this procedure the year prior.

For most procedures included in this study, we did not observe a clinically meaningful increase in same-day discharge rates throughout the study period. We hypothesize that regional and institutional variations exist in the rates of outpatient surgery for these procedures and, while the ACS-NSQIP data set precludes geographic or site-specific identification, future research using other data sources should be performed to investigate this topic. One explanation for the lack of uptake of outpatient surgery may be a reluctance on the part of surgeons to discharge patients on the day of operation for fear of missing complications. Although these concerns are well-founded, an increasing number of studies have illustrated the safety and feasibility of outpatient surgery across a variety of general surgical procedures.^1,24,25^ In addition to evidence showing the advantages of this approach, successful implementation of outpatient surgery requires buy-in from key stakeholders and development of formal patient selection criteria and perioperative care pathways. A potential avenue for achieving this could be through concerted quality improvement initiatives that include Plan-Do-Study-Act cycles to pilot outpatient surgery cases for specific procedures.

### Limitations

Our study has several important limitations, some of which are inherent in the retrospective analysis of deidentified ACS-NSQIP data. Although a large number of hospitals participate in the ACS-NSQIP, the number and nature of institutions varies each year, and patients from academic medical centers may be overrepresented in the database. Thus, the data set should not be considered a nationally representative sample, and our findings may not be generalizable to other centers in the US and internationally. Another limitation of ACS-NSQIP is that the data cannot be stratified by geographic location or facility. Furthermore, while we used multivariable logistic regression to adjust for differences in baseline characteristics, unobserved confounding is likely, and it is plausible that patients who underwent surgery during the COVID-19 pandemic may have been sicker due to case prioritization, which may have impacted the likelihood of same-day discharge. Nonetheless, with the increasing endorsement of outpatient surgery from patients, surgeons, and policy makers, our findings may help inform ongoing efforts to transition to this practice, particularly for procedures whose outpatient surgery rates remained unchanged from pre–COVID-19 levels and for which substantial case backlogs are likely to exist.

## Conclusions

This cohort study found that for most commonly performed general surgical procedures at ACS-NSQIP–participating hospitals, although the first year of the COVID-19 pandemic was associated with an increased odds of same-day discharge, a substantial increase in the proportion of outpatient cases from baseline (prepandemic) levels was observed in only 4 procedures. These findings should be used to guide studies to determine potential barriers to the uptake of outpatient surgery, particularly for procedures such as minimally invasive cholecystectomy, fundoplication, and colectomy, all of which have been shown to be safe when performed on an outpatient basis in appropriately selected patients.^1,24,25^ Further studies to determine potential complications associated with this approach and the changes in practices that occurred during subsequent surges in COVID cases in 2021 are warranted.

## Notes

Supplement 1. **eFigure.** Time Trends of Operations Without a Clinically Meaningful (≥10%) Increase in the Proportion of Outpatient Procedures Over the Study Period (2016-2020) **eTable 1.** *CPT*, *ICD-9-CM*, and *ICD-10-CM* Codes Used to Identify Patients **eTable 2.** Sensitivity Analysis of Odds of Undergoing Outpatient General Surgery Procedures During Q2-Q4 of 2020 vs Q2-Q4 of 2019

Supplement 2.
Data Sharing Statement
