# Supplementary material for: Performance of General Surgical Procedures in Outpatient Settings Before and After Onset of the COVID-19 Pandemic
Source: JAMA Netw Open. 2023 Mar 2;6(3):e231198. doi: 10.1001/jamanetworkopen.2023.1198 (PMC9982689; doi:10.1001/jamanetworkopen.2023.1198)
Supplement: Supplement 1. — eFigure. Time Trends of Operations Without a Clinically Meaningful (≥10%) Increase in the Proportion of Outpatient Procedures Over the Study Period (2016-2020) eTable 1. CPT, ICD-9-CM, and ICD-10-CM Codes Used to Identify Patients eTable 2. Sensitivity Analysis of Odds of Undergoing Outpatient General Surgery Procedures During Q2-Q4 of 2020 vs Q2-Q4 of 2019 [file jamanetwopen-e231198-s001.pdf]

Supplementary Online Content

Shariq OA, Bews KA, Etzioni DA, Kendrick ML, Habermann EB, Thiels CA. Performance of general surgical procedures in outpatient settings before and after onset of the COVID-19 pandemic. *JAMA Netw Open*. 2023;6(3):e231198. doi:10.1001/jamanetworkopen.2023.1198

eFigure. Time Trends of Operations Without a Clinically Meaningful (≥10%) Increase in the Proportion of Outpatient Procedures Over the Study Period (2016-2020)

eTable 1. *CPT*, *ICD-9-CM*, and *ICD-10-CM* Codes Used to Identify Patients

eTable 2. Sensitivity Analysis of Odds of Undergoing Outpatient General Surgery Procedures During Q2-Q4 of 2020 vs Q2-Q4 of 2019

This supplementary material has been provided by the authors to give readers additional information about their work.

© 2023 Shariq OA et al. *JAMA Network Open*.

eFigure. Time Trends of Operations Without a Clinically Meaningful (≥10%) Increase in the Proportion of Outpatient Procedures Over the Study Period (2016-2020)

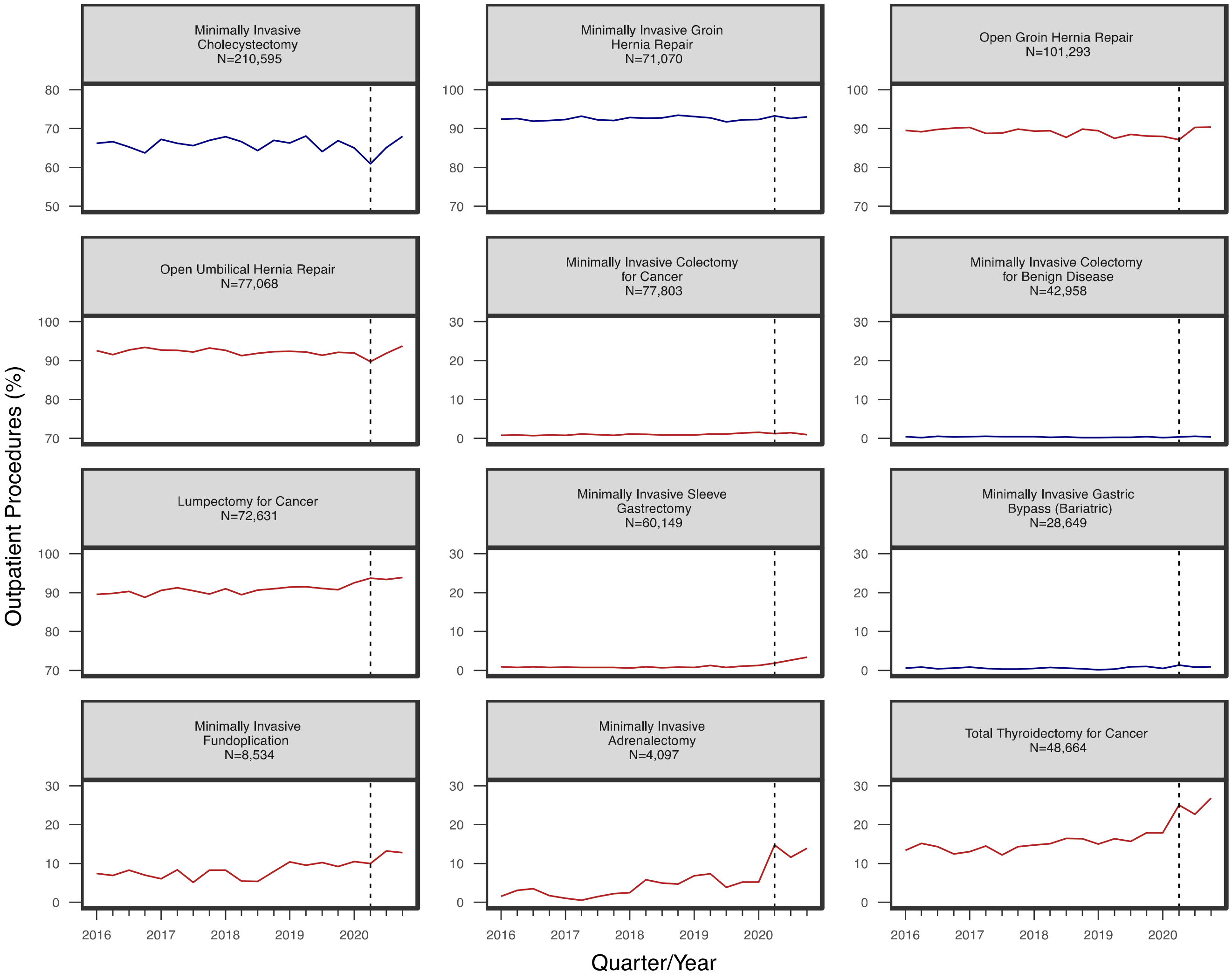

Red and blue lines indicate significant ($P < 0.05$) and non-significant ($P \geq 0.05$) trends over time, respectively, as determined by Cochrane-Armitage tests. The vertical dotted line represents quarter 2 of 2020 (the onset of the COVID-19 pandemic in the United States).

© 2023 Shariq OA et al. *JAMA Network Open*.

eTable 1. *CPT*, *ICD-9-CM*, and *ICD-10-CM* Codes Used to Identify Patients

**Procedure**	***CPT* codes**	***ICD-9-CM* codes***	***ICD-10-CM* codes**
Minimally invasive colectomy for cancer	44204, 44205, 44206, 44207, 44208	153, 171.5, 195.2, 197.5, 197.6, 198.89, 199.1, 209.1, 209.11, 209.12, 209.13, 209.14, 209.15, 209.16, 209.17, 209.20, 209.27, 209.30, 209.5, 209.51, 209.52, 209.53, 209.54, 209.55, 209.56, 209.57, 209.60, 230.3, 230.4, 235.2, 235.5, 238.9, 239.0	C7A.00, C7A.02, C7A.020, C7A.021, C7A.022, C7A.023, C7A.024, C7A.025, C7A.026, C7A.029, C7A.096, C7A.1, C7A.8, C18, C18.x, C19.x, C20.x, C21.x, C49.A0, C49.A4, C49.A5, C76.2, C78.5, C78.6, C79.89, C80.1, D01.0, D01.1, D01.2, D3A.00, D3A.020, D3A.021, D3A.022, D3A.023, D3A.024, D3A.025, D3A.026, D3A.029, D3A.096, D12.x, D37.3, D37.4, D37.5, D37.9, D48.9, D49.0
Minimally invasive colectomy for benign disease	44204, 44205, 44206, 44207, 44208		K50.x, K51.x, K52.x, K57.x, K58.x, K59.x
Lumpectomy for breast cancer	19301, 19302		C50.x, D05.x
Mastectomy for breast cancer	19303, 19305, 19306, 19307		C50.x, D05.x
Minimally invasive inguinal hernia repair	49650, 49651, 49659		K40.x, K41.x
Open inguinal hernia repair	49507, 49520, 49521, 49525, 49505, 49550, 49553, 49555, 49557		K40.x, K41.x
Minimally invasive ventral hernia repair	49652, 49653, 49654, 49655, 49656, 49657		K42.x, K43.x, K46.x
Open umbilical hernia repair	49585, 49587		K42.x
Minimally invasive sleeve gastrectomy	43775	278, 278.01	E66, E66.0, E66.01, E66.09, E66.1, E66.2, E66.8, E66.9, E88.81, K95.89, Z68.41, Z68.42, Z68.43, Z68.44, Z98.84
Minimally invasive gastric bypass	43644	278, 278.01	E66, E66.0, E66.01, E66.09, E66.1, E66.2, E66.3, E66.8, E66.9, E88.81, Z68.41, Z68.42, Z68.43, Z98.84

© 2023 Shariq OA et al. *JAMA Network Open*.

Minimally invasive cholecystectomy	47562, 47563		K80.x, K81.x, K82.x
Minimally invasive fundoplication	43280		K21.x, K44.x
Parathyroidectomy	60500, 60502	227.1, 252, 252.01	D35.1, E21.x, E83.52, N25.81
Thyroid lobectomy	60200, 60210, 60220	193, 237.4, 239.7, 234.8, 199.1, 258.02	C73, C80.1, D44.x, D49.7, D09.3, E04.x, E05.x, E06.x, E07.x, E31.22
Total thyroidectomy	60240, 60252, 60254, 60260, 60271		C73, C80.1, D09.3, D44.x, D49.7, E04.x, E05.x, E06.x, E07.x, E31.22
Minimally invasive adrenalectomy	60650	194, 194.0, 198.7, 237.2, 239.7	C74, C74.00, C74.01, C74.02, C74.10, C74.11, C74.12, C74.91, C74.92, C79.7, C79.70, C79.71, C79.72, D35.x, D44.1, D44.10, D44.11, D44.12, D49.7, E24.x, E26.x, E27.x

ICD-9-CM, International Classification of Diseases, Clinical Modification, Ninth Revision; ICD-10-CM, International Classification of Diseases, Clinical Modification, Tenth Revision, CPT, Current Procedural Terminology

*Although ICD-10-CM was implemented in the ACS-NSQIP Participant User File from 2016, six procedures (minimally invasive colectomy for cancer, minimally invasive sleeve gastrectomy, minimally invasive gastric bypass, parathyroidectomy, thyroid lobectomy, and minimally invasive adrenalectomy) additionally utilized ICD-9-CM diagnosis codes in 2016. In order to capture these procedures, both ICD-9-CM and ICD-10-CM codes were used for these six procedures in 2016 only. For the years 2017-2020 only ICD-10-CM codes were used for all procedures.

© 2023 Shariq OA et al. *JAMA Network Open*.

eTable 2. Sensitivity Analysis of Odds of Undergoing Outpatient General Surgery Procedures During Q2-Q4 of 2020 vs Q2-Q4 of 2019

**Procedure**	**Years**	**Univariate odds ratio (95% CI)**	***P* value**	**Adjusted odds ratio (95% CI)[a]**	***P* value**
Minimally invasive colectomy for cancer	Q2-Q4 2020 vs Q2-Q4 2019	1.01 (0.79-1.28)	0.97	1.01 (0.79-1.29)	0.92
Lumpectomy for cancer	Q2-Q4 2020 vs Q2-Q4 2019	1.44 (1.30-1.60)	<0.001	1.44 (1.30-1.60)	<0.001
Mastectomy for cancer	Q2-Q4 2020 vs Q2-Q4 2019	2.87 (2.65-3.11)	<0.001	2.94 (2.71-3.18)	<0.001
Minimally invasive adrenalectomy	Q2-Q4 2020 vs Q2-Q4 2019	2.63 (1.75-3.96)	<0.001	2.53 (1.67-3.83)	<0.001
Thyroid lobectomy for cancer	Q2-Q4 2020 vs Q2-Q4 2019	1.55 (1.42-1.70)	<0.001	1.54 (1.40-1.69)	<0.001
Minimally invasive inguinal hernia repair	Q2-Q4 2020 vs Q2-Q4 2019	1.10 (0.99-1.22)	0.07	1.11 (0.99-1.23)	0.06
Open inguinal hernia repair	Q2-Q4 2020 vs Q2-Q4 2019	1.16 (1.07-1.26)	<0.001	1.17 (1.08-1.27)	<0.001
Minimally invasive ventral hernia repair	Q2-Q4 2020 vs Q2-Q4 2019	1.23 (1.16-1.30)	<0.001	1.24 (1.17-1.31)	<0.001
Open umbilical hernia repair	Q2-Q4 2020 vs Q2-Q4 2019	1.04 (0.93-1.15)	0.53	1.04 (0.93-1.16)	0.52
Minimally invasive sleeve gastrectomy	Q2-Q4 2020 vs Q2-Q4 2019	2.82 (2.02-3.93)	<0.001	2.81 (2.01-3.93)	<0.001
Minimally invasive gastric bypass	Q2-Q4 2020 vs Q2-Q4 2019	1.27 (0.69-2.35)	0.45	1.23 (0.66-2.30)	0.52
Parathyroidectomy	Q2-Q4 2020 vs Q2-Q4 2019	1.27 (1.16-1.40)	<0.001	1.30 (1.19-1.44)	<0.001
Minimally invasive cholecystectomy	Q2-Q4 2020 vs Q2-Q4 2019	0.93 (0.90-0.96)	<0.001	0.93 (0.90-0.97)	<0.001
Minimally invasive fundoplication	Q2-Q4 2020 vs Q2-Q4 2019	1.32 (1.00-1.73)	0.05	1.30 (0.98-1.71)	0.07
Minimally invasive colectomy for benign disease	Q2-Q4 2020 vs Q2-Q4 2019	1.36 (0.76-2.44)	0.30	1.51 (0.83-2.73)	0.18
Total thyroidectomy for cancer	Q2-Q4 2020 vs Q2-Q4 2019	1.65 (1.51-1.79)	<0.001	1.67 (1.53-1.82)	<0.001

Abbreviations: CI, confidence interval
[a]Covariates adjusted for in multivariable logistic regression models included: American Society of Anesthesiologists class, age, smoking status, sex, and body mass index.

© 2023 Shariq OA et al. *JAMA Network Open*.
