# Supplementary material for: Performance of General Surgical Procedures in Outpatient Settings Before and After Onset of the COVID-19 Pandemic
Source: JAMA Netw Open. 2023 Mar 2;6(3):e231198. doi: 10.1001/jamanetworkopen.2023.1198 (PMC9982689; doi:10.1001/jamanetworkopen.2023.1198)
Supplement: Supplement 2. — Data Sharing Statement [file jamanetwopen-e231198-s002.pdf]

Data Sharing Statement

Shariq. Performance of General Surgical Procedures in Outpatient Settings Before and After Onset of the COVID-19 Pandemic. *JAMA Netw Open*. Published March 02, 2023. doi:10.1001/jamanetworkopen.2023.1198

Data
Data available: No

Additional Information
Explanation for why data not available: The national database used in this study does not allow for this however participating institutions can repeat the analysis using the same methodology as in the manuscript.
